# Evaluation of Treatment Effect with Paired Failure Times in a Single-Arm Phase II Trial in Oncology

**DOI:** 10.1155/2018/1672176

**Published:** 2018-01-11

**Authors:** Matthieu Texier, Federico Rotolo, Michel Ducreux, Olivier Bouché, Jean-Pierre Pignon, Stefan Michiels

**Affiliations:** ^1^Biostatistics and Epidemiology Unit, Gustave Roussy, Université Paris-Saclay, 94805 Villejuif, France; ^2^CESP INSERM U1018, Paris-Sud University, 94805 Villejuif, France; ^3^Gastrointestinal Oncology Unit, Gustave Roussy, Université Paris-Saclay, 94805 Villejuif, France; ^4^Gastrointestinal Unit, University Hospital, Reims, France

## Abstract

In early phase clinical trials of cytotoxic drugs in oncology, the efficacy is typically evaluated based on the tumor shrinkage. However, this criterion is not always appropriate for more recent cytostatic agents, and alternative endpoints have been proposed. The growth modulation index (GMI), defined as the ratio between the times to progression in two successive treatment lines, has been proposed for a single-arm phase II trials. The treatment effect is evaluated by estimating the rate of patients having a GMI superior to a given threshold. To estimate this rate, we investigated a parametric method based on the distribution of the times to progression and a nonparametric one based on a midrank estimator. Through simulations, we studied their operating characteristics and the impact of different design parameters (censoring, dependence, and distribution) on them. In these simulations, the nonparametric estimator slightly underestimated the rate and had slightly overconservative confidence intervals in some cases. Conversely, the parametric estimator overestimated the rate and had anticonservative confidence intervals in some cases. The nonparametric method appeared to be more robust to censoring than the parametric one. In conclusion, we recommend the nonparametric method, but the parametric method can be used as a supplementary tool.

## 1. Introduction

In oncology, if a new treatment is found to be acceptably safe in a phase I clinical trial, it can be tested in a phase II trial to look for evidence of efficacy. The type of response or benefit to evaluate depends on the goals of the treatment; in advanced cancer trials, the most used endpoints are related to the change of the size of the lesion or its disappearance. Historically, the tumor shrinkage was the primary endpoint in phase II trials for cytotoxic cancer drugs. Since the 90s, cytostatic drugs, which are supposed to modulate the tumor growth without causing immediate shrinkage, are being developed. Thus, Von Hoff [1] and Mick et al. [2] advocated for rather evaluating the time to progression (TTP) as the primary endpoint in a one-stage design. Since patients being offered phase II studies of new agents have typically failed a previous regimen, then all first progressions are observed and TTP before experimental treatment, say TTP_1_, is known for all the patients enrolled. Conversely, the TTP after the experimental agent, TTP_2_, may or may not be censored at the time of the analysis. As the TTP is highly variable across patients and the degree of correlation between the paired failure times is a key feature, Von Hoff [1] proposed to evaluate the growth modulation index (GMI = TTP_2_/TTP_1_) instead, so that each patient serves as his/her own historical control. Von Hoff [1] assumed a null ratio value of 1 and that the GMI needs to be greater than 1.33 for a new regimen to be considered effective in delaying progression. Mick et al. [2] argued that because patients enter a new treatment line after a new progression, the prognosis is expected to be poorer than at the previous treatment line. Thus, because of the natural history of the disease, one expects that in general TTP_2_ is shorter than TTP_1_, which would indicate a null ratio value smaller than 1 and that a GMI superior to 1 is enough for considering a new regimen as effective.

Some authors have started employing the GMI as primary endpoint. At the time of writing (April 2017), there were a total of ten oncology trials registered in the European Union Clinical Trial Register and eleven oncology trials registered on the https://www.clinicaltrials.gov website as using GMI. For example, Von Hoff et al. [3] used the GMI to measure the activity of a targeted therapy selected by molecular profiling in patients having failed all effective treatments. Eighteen out of 66 patients (27%) had a progression-free survival (PFS) ratio superior to 1.33 (95% confidence interval: [17%; 38%]). Several others published trials [4–6] used a GMI-based approach to assess the activity of second-line treatments, but the estimation did not account for patients with censored times to progression. Only a recent secondary analysis of the SHIVA trial estimated the PFS ratio by Kaplan-Meier curves [7].

Before the GMI can be regularly used as primary endpoint in phase II studies, we need appropriate statistical methods and detailed knowledge of its statistical characteristics. In the present paper, we present methods to estimate the proportion of patients having a GMI greater than a given threshold by handling censored observations, we explore their operating characteristics via simulations and we show an application on a real data set. Such a motivating study in advanced colorectal cancer is presented in Section 2.  Section 3 summarizes the statistical methodology to estimate the probability that the GMI is higher than a given threshold.  Section 4 presents a simulation study to investigate parameters which could influence the performance of the estimators. Finally, in Section 5, we apply the presented methods to real data.  Section 6 discusses the findings.

## 2. Motivating Example

**Figure 1:**
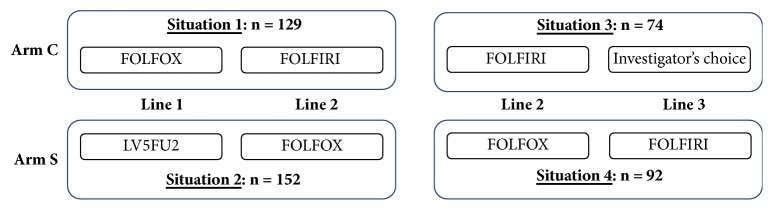
Single-arm scenarios based on the FFCD 2000-05 trial.

The FFCD 2000-05 trial [8, 9] was a randomized trial conducted by the French Federation of Digestive Oncology, which included 410 patients with advanced colorectal cancer. It was a phase III trial comparing a sequential (S) arm to a combination (C) arm. Patients in arm S were treated with 5-fluorouracil and leucovorin (LV5FU2) in first line, then with FOLFOX (LV5FU2 + oxaliplatin) in second line, and then with FOLFIRI (LV5FU2 + irinotecan) in third line. Patients in arm C were treated directly with FOLFOX in first line and then with FOLFIRI in second line. The times to progression in the first, second, and third treatment lines were recorded for patients who entered each line of treatment, respectively. Such a design provided us with four separate scenarios in which the effect of the treatment between each couple of lines can be estimated (Figure 1). We considered line 2 versus line 1 in arm C (FOLFOX versus FOLFIRI) as representative of a phase II framework. Then, we compared results to those obtained considering line 3 versus line 2 in arm S (FOLFOX versus FOLFIRI, again), which contrasts the same drugs, despite the fact that patients had been treated previously by LV5FU2 alone.

## 3. Methods

### 3.1. Dependence between TTP_1_ and TTP_2_

The time to progression (TTP) is likely to be linked to general characteristics of each patient, whatever the treatment line. Because TTP_1_ and TTP_2_ share these common factors, Von Hoff [1] expected that the growth modulation index (GMI) is a less heterogeneous endpoint, as some of the variability of TTP_2_ may be captured through TTP_1_. Therefore, the correlation between successive times to progression could play a key role in determining the performance of the GMI as clinical endpoint. Mick et al. [2] showed, through simulations, that reasonable power for a trial was only attainable for moderate to strong correlation between consecutive times to progression.

As the dependence between TTP_1_ and TTP_2_ is due to some underlying factors shared by the two time-to-event variables, it can be modeled in a very natural way via shared frailty models [10]. The shared frailty model is an extension of the proportional hazards model in which an unobservable random quantity, called the frailty term, acts multiplicatively on the baseline hazard functions of the time variables. This term accounts for intrapatient correlation. The frailty model is defined in terms of the conditional hazard:

(1) $$\begin{array}{c} h_{ji}\left(\;t\;|\;u_{i}\;\right)=h_{j0}\left(\;t\;\right)u_{i}\exp\left(\;x_{ji}^{T}\beta_{j}\;\right), \end{array}$$

for patient *i* ∈ {1,…, *n*} at treatment line *j* ∈ {1,2}, and where *h*_*j*0_(*t*) is the treatment line-specific baseline hazard function, *u*_*i*_ the frailty term for the patient *i*, *x*_*ji*_ the vector of his/her covariates in the *j*th treatment line, and *β*_*j*_ the vector of regression coefficients. In a gamma frailty model, the frailty term is a random variable with probability density function:

(2) $$\begin{array}{c} f\left(\;u\;\right)=\frac{\theta^{-1/\theta }u^{1/\theta -1}\exp\left(-u/\theta \right)}{\Gamma \left(1/\theta \right)}, \end{array}$$

where Γ(·) is the gamma function. This distribution corresponds to a gamma distribution with mean and variance equal to 1 and *θ*. Shared frailty models allow estimating the intrapatient dependence via Kendall's *τ*, which is a rank correlation measure of the concordance between time pairs. In the case of a gamma frailty model, Kendall's *τ* is equal to *θ*/(*θ* + 2) and can thus be estimated by plugging in the estimate of *θ*. Different distributions can be assumed for the baseline hazard [11]; we chose a Weibull distribution because it was the one which fitted the best our advanced colorectal data. We fitted and compared the parametric frailty models using the parfm package in R [11].

### 3.2. Growth Modulation Index TTP_2_/TTP_1_

If we consider a study in which patients enter after having a first progression, the time to progression at prior therapy (TTP_1_) is always observed by design. After a first progression, the experimental treatment is administered. Contrary to TTP_1_, the time to progression with the new therapy (TTP_2_) can be right-censored. In that case, also the growth modulation index GMI = TTP_2_/TTP_1_ [1] is right-censored. As this ratio is a nonnegative and possibly right-censored random variable, it can be treated as a time-to-event variable [12]. Therefore, the statistic of interest,

(3) $$\begin{array}{c} S_{GMI}\left(\;\delta \;\right)=P\left[\;\frac{{TTP}_{2}}{{TTP}_{1}}>\delta \;\right],\;\delta \geq 0, \end{array}$$

can be handled as the survival probability of a time-to-event random variable at a given time point *δ*. For a given threshold *δ*, we define a patient as “responder” if his/her GMI is greater than *δ* and “nonresponder” otherwise. Since, in advanced cancer patients, successive TTPs tend to be shorter and shorter [13], GMI ≥ 1 should be considered as a sign of drug activity, which is less conservative than the threshold *δ* = 1.33 proposed by Von Hoff [1]. In what follows, we describe two methods, a parametric and a nonparametric one, to estimate *S*_GMI_(*δ*) for any choice of *δ*.

#### 3.2.1. Nonparametric Method

The nonparametric approach, inspired by the Wilcoxon rank sum test, consists in using the ranks of each pair (TTP_1_, TTP_2_) to estimate *S*_GMI_(*δ*). Due to censoring, the ranks of some observations are unknown but can be estimated by midranks. Midranks are computed according to the procedure proposed by Hudgens and Satten [14] which can be summarized as follows.

For each patient *i* = 1,…, *n*, the pair of times (TTP_1*i*_; TTP_2*i*_) is observed. Each time TTP_*ji*_  (*j* = 1,2) is decomposed into an interval, denoted [*L*_*ji*_; *R*_*ji*_]. The left bound is always fixed to *L*_*ji*_ = TTP_*ji*_. If TTP_*ji*_ is observed (which is always the case for *j* = 1) then *R*_*ji*_ = TTP_*ji*_. If TTP_*ji*_ is right-censored (which is only possible for *j* = 2), then *R*_2*i*_ = *∞*. The midranks are computed using the minimum and the maximum ranks of the interval bounds associated with each TTP_*ji*_ as follows. Given TTP_*ji*_, the minimum rank is the rank of *L*_*ji*_ among the 2*n* pooled *R*_*j*_'s:

(4) minji:Rj1≤Rj2≤⋯≤Rjmini−1≤Lji≤Rjmini≤⋯≤Rj2n.

The maximum rank is the rank of *R*_*ji*_ among the 2*n* pooled *L*_*ji*_'s:

(5) maxji:Lj1≤Lj2≤⋯≤Ljmaxi≤Rji≤Ljmaxi+1≤⋯≤Lj2n.

Now, the midrank *M*_*ji*_ is the midpoint of the minimum and the maximum rank:

(6) $$\begin{array}{c} M_{ji}=\frac{{min}_{ji}+{max}_{ji}}{2}. \end{array}$$

To estimate *S*_GMI_(*δ*), we replace TTP_1*i*_ with TTP′_1*i*_ = *δ*TTP_1*i*_ and compute the midranks *M*′_1*i*_ of TTP′_1*i*_ and *M*_2*i*_ of TTP_2*i*_ to obtain the *n* pairs of midranks (*M*′_1*i*_; *M*_2*i*_). Finally, the estimate of the probability of interest is as follows:

(7) $$\begin{array}{c} {\hat{S}}_{GMI}\left(\;\delta \;\right)=\frac{1}{n}\overset{n}{\underset{i=1}{\sum }}I\left(\;M_{2i}\geq {M^{\prime }}_{1i}\;\right), \end{array}$$

with *I*(·) being the indicator function which takes value 1 if its argument is true and 0 otherwise.

#### 3.2.2. Parametric Method

In this approach, a parametric probability distribution is assumed for the GMI, so that the probability of interest can be easily derived as a function of the estimated distribution parameters. Let us assume that, conditionally on a frailty term *u*_*i*_, TTP_1_ and TTP_2_ have Weibull marginal distributions *W*(*a*; *b*_1_*u*_*i*_) and *W*(*a*; *b*_2_*u*_*i*_) with a common shape parameter *a*:

(8) fjx;a,bj ∣ ui=auibj−axa−1exp⁡−xuibja.

Then, Owen [15] showed that the ratio TTP_2_/TTP_1_ follows a log-logistic distribution,

(9) $$\begin{array}{c} f\left(\;\delta ;a,\kappa \;\right)=a\kappa^{a}\delta^{a-1}{\left(\;1+{\left(\;\delta \kappa \;\right)}^{a}\;\right)}^{-2},\;\delta \geq 0, \end{array}$$

with *κ* = *b*_1_/*b*_2_, which does no longer depend on the shared frailty *u*_*i*_.

By using this distribution, we can obtain maximum likelihood estimates of the distribution parameters and directly derive the probability of interest from the survival function:

(10) $$\begin{array}{c} S\left(\;\delta ;\hat{\alpha },\hat{\kappa }\;\right)={\left(\;1+{\left(\;\delta \hat{\kappa }\;\right)}^{\hat{a}}\;\right)}^{-1}. \end{array}$$

Parameter estimates were computed using the survreg function in the R package survival.

R code of the two methods is available for download on https://github.com/Oncostat/TTPratio.

## 4. Simulation Study

### 4.1. Simulation Design

We designed a simulation study to evaluate the influence of the design parameters on the two estimators of *S*_GMI_(*δ*). We varied (i) the dependence between the two successive times to progression via Kendall's *τ*, (ii) the shape *a* of the distribution of TTP_*j*_, (iii) the relative effect *e* of the second-line treatment as compared to the first-line treatment, and (iv) the censoring rate *r* for TTP_2_.

#### 4.1.1. Data Generation

First, for given values of the parameters of interest, we generated a frailty term *u*_*i*_ for each patient using random values from a gamma distribution with density given in Section 3. Due to the link between *τ* and *θ*, for a given *τ*, we could fix *θ* = 2*τ*/(1 − *τ*). Three values of *τ* were used in our simulation: 0.1, 0.2, and 0.3.

Then, we generated times to first and second progressions from Weibull distribution with density:

(11) fjx;a,bj ∣ ui=auibj−axa−1exp⁡−xuibja,j=1,2.

For the shape parameter *a*, common to the two distributions, we considered three values: 0.5, 1, and 2. A shape of *a* = 0.5 represents a metastatic disease with a median of TTP_1_ greater than 15 months, whereas a shape of *a* = 2 corresponds to a more aggressive disease (median of TTP_1_ close to 6 months).

The scale parameter was different for the two distributions: *b*_2_ = *b*_1_*∗e*, where *e* is the median of TTP_2_/TTP_1_. We considered three values for *e*: 0.77, representing inactivity of the second-line treatment; 1, representing an equivalence of the two treatments; and 1.33, representing efficacy according to the definition of Von Hoff [1].

Independent and noninformative censoring was introduced by taking the minimum between TTP_2_ and a random uniform variable. Desired censoring rates (10% and 40%) were obtained by controlling the support of the uniform distribution.

We performed 10,000 simulations for each of the 54 scenarios defined by *a*, *e*, *τ*, and a censoring rate. The statistical properties of the parametric and nonparametric estimators were evaluated in terms of the mean bias, the average standard error, and the empirical standard error, the latter being defined as the standard deviation of the 10,000 estimates.

### 4.2. Results

**Figure 2:**
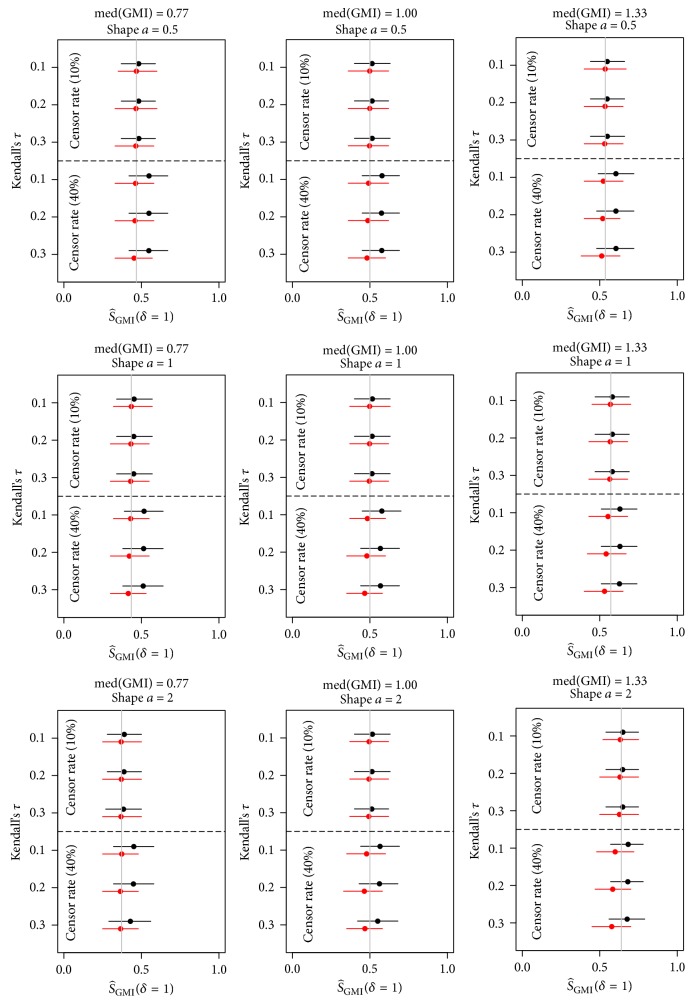
Probability ${\hat{S}}_{GMI}\left(\delta =1\right)$ of GMI being greater than 1 estimated in the simulation study via the parametric (black) and nonparametric (red) methods. Normally approximate 95% confidence intervals using the empirical standard error.

The results of the simulations are summarized in Figure 2 (see Supplementary Tables A1–A6 for detailed results). The nonparametric method underestimated the probability of interest in 51/54 scenarios, but the mean bias was low in general, ranging across scenarios from −0.062 to 0.001 (median: −0.006). On the contrary, the parametric method always overestimated the probability of interest, but the mean bias was low as well, ranging across scenarios from 0.009 to 0.082 (median: 0.028). With a censoring rate of 10% and considering all scenarios, the nonparametric estimator was slightly less biased than the parametric estimator (median absolute bias: 0.003 versus 0.014): the absolute bias of the nonparametric estimator was at most 0.011 and the bias of the parametric estimator was at most 0.018. The bias of the parametric estimator increased with increasing censoring rate; across all scenarios with censoring rate of 40%, its median absolute bias was 0.069. The nonparametric estimator was more robust to censoring with a median absolute bias of 0.018 for 40% of censoring.

Both estimators were robust to changes in dependence, shape parameter *a*, and treatment effect *e*. Considering all scenarios, the average (over the 10,000 replicates) of the estimated standard error (ASE) via the nonparametric method was greater than or equal to the empirical standard error (ESE). This suggests that the nonparametric confidence intervals are more conservative than their nominal level. For the parametric estimator, on the contrary, when we considered second-line treatment inactivity (median GMI = 0.77) and 40% of censoring, the ASE was smaller than the ESE. This means that parametric confidence intervals are too liberal under the null hypothesis.

## 5. Application to the FFCD 2000-05 Trial

In this section, we illustrate the presented methodology to the data of the FFCD2000-05 trial (see Section 2 and Figure 1). As discussed previously, we will consider situations 1 and 4 only, in which the same couple of treatments are contrasted. The ratio TTP_2_/TTP_1_ could be evaluated on 129 patients in situation 1. The ratio TTP_3_/TTP_2_ could be evaluated on 92 patients in situation 4. A total of 15 patients (12%) had their TTP_2_ censored in situation 1 and 13 patients (14%) had their TTP_3_ censored in situation 4.

### 5.1. Dependence between TTP1 and TTP2

As discussed in Section 3, we estimated Kendall's *τ* by modeling the risks of progression via shared frailty models. Weibull distributions were assumed for the baseline hazard functions. The use of a gamma distribution for the frailty term was justified by a preliminary study comparing the Akaike Information Criterion (AIC) of the model with gamma and inverse Gaussian frailty distributions. The positive stable frailty distribution was considered too, but it was also discarded due to the lack of numerical convergence. In all four situations, the model with gamma distribution had the smallest AIC.

In situation 1, the estimated Kendall's *τ* was 0.195, a relatively low correlation. In situation 4, that is FOLFOX versus FOLFIRI again, but after a first line with LV5FU2, the estimated Kendall's *τ* was slightly higher: 0.225. Even weaker dependence was estimated for situation 3 (*τ* = 0.152) and situation 2 (*τ* = 0.142). Overall, these values fell in between the first and second values of *τ* considered in our simulations: 0.1 and 0.2.

### 5.2. Estimation of ${\hat{S}}_{GMI}(\delta )$

**Figure 3:**
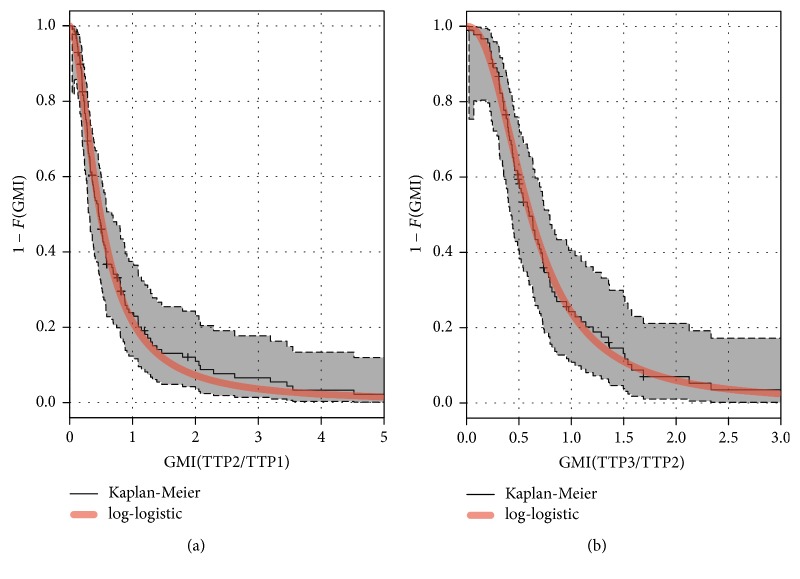
Survival function estimate of the growth modulation index (situation 1 in (a); situation 4 in (b)) via the Kaplan-Meier method and via a log-logistic distribution. The gray area is the 95% confidence band for the Kaplan-Meier estimate.

To apply the parametric estimation method for *S*_GMI_(1) described in Section 3, we assumed Weibull distributions of times to progression with common shape parameter. This assumption was needed in order to assume a log-logistic distribution for their GMI. Thus, we fitted the Kaplan-Meier estimates of the GMI and compared them to the maximum likelihood log-logistic survival curves to informally check the appropriateness of the parametric assumption.  Figure 3 shows, for situations 1 and 4, the Kaplan-Meier estimates of the GMI with the estimated log-logistic survival curves. This distribution seems to fit quite well to the data.

In situation 1, the estimated probability that the GMI ≥1 was ${\hat{S}}_{GMI}\left(1\right)$ = 0.21 with the parametric estimator (95% Confidence interval: [0.14; 0.29]) and ${\hat{S}}_{GMI}\left(1\right)$ = 0.24 with the nonparametric estimator (95% CI: [0.17; 0.31]). In situation 4, comparing the same two treatments after an LV5FU2 line, the estimated probability was 0.24 (95% CI: [0.15; 0.33]) with the parametric estimator and 0.27 (95% CI: [0.18; 0.36]) with the nonparametric estimator. These results suggest that the sequence “FOLFOX in first line/FOLFIRI in second line” leads to a shortened time to progression: FOLFIRI's activity in second line seems inferior to FOLFOX's activity in first line.

Table 1 shows the different estimations for the other situations, too. The activity of FOLFOX in second line seems to be comparable to the activity of LV5FU2 in first line for patients in the arm S.

## 6. Discussion

The growth modulation index (GMI) is more and more used to evaluate the treatment effect in single-arm phase II trials. An increasing number of clinical trials employ the GMI and the European Medicine Agency (EMA), in its “Guideline on Evaluation of Anticancer Medicinal Products in Man,” admits its utilization for a comparison between two successive therapies [16]. By choosing an adequate threshold *δ* (0.77, 1, or 1.33), the estimated probability of interest *S*_GMI_(*δ*) is a practical measure of the proportion of patients for whom two successive lines of treatment are ineffective, equivalent, or effective.

In this article, we evaluated two ways to estimate *S*_GMI_(*δ*) and we investigated how the design parameters had an impact on these estimators. The censoring rate had an impact on the parametric and nonparametric estimators, respectively. In our simulations, the nonparametric method was more robust to high censoring rates, but the average bias was small in any case. Thus, the use of this method in phase II studies could represent substantial time savings for the analysis when the disease in question progresses slowly over time. Von Hoff [1] showed the key role of dependence between the paired times to progression, but in our study this parameter did not have a noticeable impact neither on the bias nor on the empirical standard error. The few published clinical trials that used the GMI as a criterion of activity reported a rather low correlation of the paired time to progression. However, in some of them, such a low correlation may be due to the heterogeneity of the first-line treatment (different nature of chemotherapy) or to the localization of the tumor. In Penel et al. [17], for instance, the analysis did not account for the heterogeneity of the subtypes of sarcoma. Further studies are needed to detect the influence of cancer localization on the different design parameters. To date, it is not well known in which cancer types the intrapatient correlation is the strongest.

There are practical limitations to the use of GMI in a phase II study. The collection of PFS or TTP measurements for each patient has to be very precise and homogeneous between patients and, if the case, between centers. The frequency of the follow-up evaluations affects the estimation of TTP and PFS [18]. This issue should be considered carefully in the design and the conduct of a trial employing this endpoint.

In clinical practice, patients can interrupt the first-line treatment for many reasons such like toxicity occurrence. In that case, they can enter the second line without a progression, causing TTP_1_ being censored. For these patients, TTP_2_/TTP_1_ is left censored (the GMI is unknown but an upper bound is known) and inferential methods can be adapted to that situation. Nevertheless, if both TTP_1_ and TTP_2_ are censored, neither an upper nor a lower bound is known and the observation is noninformative. However, one could argue that phase II studies using GMI as the primary endpoint should enroll only patients who have failed previous treatment and thus exclude cases where TTP_1_ is censored. A third approach would be to consider also treatment interruptions due to toxicity as events in a treatment-failure perspective. Eventually, the most appropriate approach will depend on clinical considerations about whether the new treatment is intended for patients recurring only, or for any interruptions of the previous treatment, whatever the cause.

In our simulations, nonparametric and parametric methods, when biased, had biases in opposite directions. We recommend using the nonparametric method to estimate the proportion of patients having a GMI superior to a threshold because it is more conservative. Nevertheless, the parametric method can more easily deal with interval censoring, which is an inherent issue with progression-free survival data [19]. Consequently, the parametric method can be used as a supplementary tool.

## Figures and Tables

**Table 1:** Estimation of *S*_GMI_(*δ* = 1) = *P*(GMI > 1) for the four situations in the FFCD 2000-05 trial.

	Treatment		*N*	Events	Estimator	
	Line 1	Line 2			Parametric	Nonparametric
Arm C						
Situation 1	FOLFOX	FOLFIRI	129	114	0.21 [0.14; 0.29]	0.24 [0.17; 0.31]
Situation 3	FOLFIRI	Investigator	74	59	0.52 [0.41; 0.63]	0.54 [0.43; 0.65]
Arm S						
Situation 2	LV5FU2	FOLFOX	152	122	0.54 [0.46; 0.62]	0.48 [0.40; 0.56]
Situation 4	FOLFOX	FOLFIRI	92	79	0.24 [0.15; 0.33]	0.27 [0.18; 0.36]

## Supplementary Materials

Supplementary Materials Online-only Supplementary Materials detail the numerical results for each scenario of the simulation study. Mean bias, average standard error, and empirical standard error of nonparametric and parametric estimation are presented in Tables A1–A6. Table A1: bias, average standard error, and empirical standard error of the nonparametric estimator of the probability in *S*_GMI_(*δ* = 1) with equivalent treatments (median (GMI) = 1). Table A2: bias, average standard error, and empirical standard error of the parametric estimator of the probability *S*_GMI_(*δ* = 1) with equivalent treatments (median (GMI) = 1). Table A3: bias, average standard error, and empirical standard error of the nonparametric estimator of the probability *S*_GMI_(*δ* = 1) with an inactive second-line treatment (median (GMI) = 0.77). Table A4: bias, average standard error, and empirical standard error of the parametric estimator of the probability *S*_GMI_(*δ* = 1) with an inactive second-line treatment (median (GMI) = 0.77). Table A5: bias, average standard error, and empirical standard error of the nonparametric estimator of the probability *S*_GMI_(*δ* = 1) with an active second-line treatment (median (GMI) = 1.33). Table A6: bias, average standard error, and empirical standard error of the parametric estimator of the probability *S*_GMI_(*δ* = 1) with an active second-line treatment (median (GMI) = 1.33).
