# Supplementary material for: Evaluation of Treatment Effect with Paired Failure Times in a Single-Arm Phase II Trial in Oncology
Source: Comput Math Methods Med. 2018 Jan 11;2018:1672176. doi: 10.1155/2018/1672176 (PMC5820554; doi:10.1155/2018/1672176)
Supplement: Supplementary Materials — Online-only Supplementary Materials detail the numerical results for each scenario of the simulation study. Mean bias, average standard error, and empirical standard error of nonparametric and parametric estimation are presented in Tables A1–A6. Table A1: bias, average standard error, and empirical standard error of the nonparametric estimator of the probability in SGMI(δ = 1) with equivalent treatments (median (GMI) = 1). Table A2: bias, average standard error, and empirical standard error of the parametric estimator of the probability SGMI(δ = 1) with equivalent treatments (median (GMI) = 1). Table A3: bias, average standard error, and empirical standard error of the nonparametric estimator of the probability SGMI(δ = 1) with an inactive second-line treatment (median (GMI) = 0.77). Table A4: bias, average standard error, and empirical standard error of the parametric estimator of the probability SGMI(δ = 1) with an inactive second-line treatment (median (GMI) = 0.77). Table A5: bias, average standard error, and empirical standard error of the nonparametric estimator of the probability SGMI(δ = 1) with an active second-line treatment (median (GMI) = 1.33). Table A6: bias, average standard error, and empirical standard error of the parametric estimator of the probability SGMI(δ = 1) with an active second-line treatment (median (GMI) = 1.33). [file 1672176.f1.docx]

**Supplementary Appendix**

|  | Median TTP1 | 15 months | 10 months | 6 months |
| --- | --- | --- | --- | --- |
| Censoring % | Kendall’s τ |  |  |  |
| 10 | 0.1 | -0.002 (0.065\|0.064) | -0.002 (0.065\|0.064) | -0.006 (0.064\|0.064) |
|  | 0.2 | -0.001 (0.064\|0.064 | -0.003 (0.065\|0.064) | -0.006 (0.064\|0.064) |
|  | 0.3 | -0.003 (0.064\|0.064) | -0.005 (0.064\|0.064) | -0.007 (0.064\|0.064) |
| 40 | 0.1 | -0.009 (0.063\|0.064) | -0.016 (0.063\|0.064) | -0.022 (0.063\|0.064) |
|  | 0.2 | -0.013 (0.063\|0.064) | -0.020 (0.063\|0.064) | -0.026 (0.062\|0.064) |
|  | 0.3 | -0.019 (0.062\|0.064) | -0.034 (0.062\|0.064) | -0.033 (0.062\|0.064) |

Table A1: Bias, Average Standard Error and Empirical Standard Error of the non-parametric estimator of the probability in $S_{GMI}\left( \delta=1 \right)$ with equivalent treatments (median (GMI) = 1).

|  | Median TTP1 | 15 months | 10 months | 6 months |
| --- | --- | --- | --- | --- |
| Censoring % | Kendall’s τ |  |  |  |
| 10 | 0.1 | 0.014 (0.057\|0.064) | 0.016 (0.057\|0.064) | 0.016 (0.058\|0.064) |
|  | 0.2 | 0.014 (0.057\|0.064) | 0.014 (0.058\|0.064) | 0.013 (0.058\|0.064) |
|  | 0.3 | 0.014 (0.057\|0.064) | 0.013 (0.058\|0.064) | 0.012 (0.058\|0.064) |
| 40 | 0.1 | 0.078 (0.061\|0.064) | 0.077 (0.062\|0.063) | 0.065 (0.064\|0.063) |
|  | 0.2 | 0.074 (0.061\|0.063) | 0.067 (0.063\|0.063) | 0.061 (0.063\|0.064) |
|  | 0.3 | 0.076 (0.062\|0.063) | 0.068 (0.062\|0.064) | 0.050 (0.067\|0.064) |

Table A2: Bias, Average Standard Error and Empirical Standard Error of the parametric estimator of the probability $S_{GMI}\left( \delta=1 \right)$ with equivalent treatments (median (GMI) = 1).

|  | Median TTP1 | 15 months | 10 months | 6 months |
| --- | --- | --- | --- | --- |
| Censoring % | Kendall’s τ |  |  |  |
| 10 | 0.1 | 0.0004 (0.065\|0.064) | -0.0002 (0.064\|0.063) | -0.003 (0.062\|0.062) |
|  | 0.2 | -0.002 (0.064\|0.064) | -0.003 (0.064\|0.063) | -0.002 (0.062\|0.062) |
|  | 0.3 | -0.003 (0.065\|0.064) | -0.004 (0.064\|0.063) | -0.004 (0.062\|0.062) |
| 40 | 0.1 | -0.005 (0.062\|0.064) | -0.004 (0.062\|0.063) | 0.0009 (0.060\|0.062) |
|  | 0.2 | -0.009 (0.062\|0.064) | -0.013 (0.062\|0.063) | -0.007 (0.059\|0.061) |
|  | 0.3 | -0.015 (0.062\|0.064) | -0.020 (0.062\|0.063) | -0.006 (0.061\|0.062) |

Table A3: Bias, Average Standard Error and Empirical Standard Error of the non-parametric estimator of the probability $S_{GMI}\left( \delta=1 \right)$ with an inactive second line treatment (median (GMI) = 0.77).

|  | Median TTP1 | 15 months | 10 months | 6 months |
| --- | --- | --- | --- | --- |
| Censoring % | Kendall’s τ |  |  |  |
| 10 | 0.1 | 0.015 (0.056\|0.064) | 0.018 (0.058\|0.064) | 0.017 (0.058\|0.062) |
|  | 0.2 | 0.015 (0.057\|0.064) | 0.016 (0.057\|0.064) | 0.016 (0.057\|0.062) |
|  | 0.3 | 0.016 (0.058\|0.064) | 0.015 (0.057\|0.064) | 0.012 (0.057\|0.062) |
| 40 | 0.1 | 0.082 (0.063\|0.064) | 0.082 (0.065\|0.064) | 0.079 (0.068\|0.064) |
|  | 0.2 | 0.081 (0.063\|0.064) | 0.079 (0.066\|0.064) | 0.076 (0.068\|0.064) |
|  | 0.3 | 0.081 (0.064\|0.064) | 0.077 (0.066\|0.064) | 0.057 (0.069\|0.064) |

Table A4: Bias, Average Standard Error and Empirical Standard Error of the parametric estimator of the probability $S_{GMI}\left( \delta=1 \right)$ with an inactive second line treatment (median (GMI) = 0.77).

|  | Median TTP1 | 15 months | 10 months | 6 months |
| --- | --- | --- | --- | --- |
| Censoring % | Kendall’s τ |  |  |  |
| 10 | 0.1 | -0.0005 (0.065\|0.064) | -0.002 (0.064\|0.063) | -0.006 (0.062\|0.062) |
|  | 0.2 | -0.001 (0.064\|0.064) | -0.004 (0.064\|0.063) | -0.008 (0.062\|0.062) |
|  | 0.3 | -0.004 (0.064\|0.064) | -0.006 (0.064\|0.063) | -0.011 (0.062\|0.062) |
| 40 | 0.1 | -0.012 (0.064\|0.064) | -0.018 (0.063\|0.064) | 0.040 (0.060\|0.063) |
|  | 0.2 | -0.016 (0.063\|0.064) | -0.030 (0.063\|0.064) | -0.055 (0.060\|0.063) |
|  | 0.3 | -0.023 (0.063\|0.064) | -0.040 (0.062\|0.064) | -0.062 (0.061\|0.063) |

Table A5: Bias, Average Standard Error and Empirical Standard Error of the non-parametric estimator of the probability $S_{GMI}\left( \delta=1 \right)$ with an active second line treatment (median (GMI) = 1.33).

|  | Median TTP1 | 15 months | 10 months | 6 months |
| --- | --- | --- | --- | --- |
| Censoring % | Kendall’s τ |  |  |  |
| 10 | 0.1 | 0.013 (0.056\|0.064) | 0.010 (0.056\|0.063) | 0.010 (0.054\|0.061) |
|  | 0.2 | 0.012 (0.056\|0.064) | 0.010 (0.055\|0.063) | 0.009 (0.054\|0.061) |
|  | 0.3 | 0.013 (0.056\|0.064) | 0.010 (0.056\|0.063) | 0.009 (0.055\|0.061) |
| 40 | 0.1 | 0.069 (0.059\|0.063) | 0.059 (0.057\|0.062) | 0.044 (0.054\|0.060) |
|  | 0.2 | 0.069 (0.059\|0.063) | 0.059 (0.058\|0.062) | 0.042 (0.055\|0.060) |
|  | 0.3 | 0.069 (0.060\|0.063) | 0.056 (0.059\|0.062) | 0.038 (0.058\|0.060) |

Table A6: Bias, Average Standard Error and Empirical Standard Error of the parametric estimator of the probability $S_{GMI}\left( \delta=1 \right)$ with an active second line treatment (median (GMI) = 1.33).
